# Quantifying Neighbourhood Socioeconomic Effects in Clustering of Behaviour-Related Risk Factors: A Multilevel Analysis

**DOI:** 10.1371/journal.pone.0032937

**Published:** 2012-03-12

**Authors:** Jaana I. Halonen, Mika Kivimäki, Jaana Pentti, Ichiro Kawachi, Marianna Virtanen, Pekka Martikainen, S. V. Subramanian, Jussi Vahtera

**Affiliations:** 1 Finnish Institute of Occupational Health, Helsinki, Finland; 2 Department of Epidemiology and Public Health, University College London Medical School, London, United Kingdom; 3 Department of Society, Human Development, and Health, Harvard School of Public Health, Boston, Massachusetts, United States of America; 4 Population Research Unit, Department of Social Research, University of Helsinki, Helsinki, Finland; 5 Department of Public Health, University of Turku, and Turku University Hospital, Turku, Finland; Vanderbilt University, United States of America

## Abstract

**Background:**

The extent to which neighbourhood characteristics explain accumulation of health behaviours is poorly understood. We examined whether neighbourhood disadvantage was associated with co-occurrence of behaviour-related risk factors, and how much of the neighbourhood differences in the co-occurrence can be explained by individual and neighbourhood level covariates.

**Methods:**

The study population consisted of 60 694 Finnish Public Sector Study participants in 2004 and 2008. Neighbourhood disadvantage was determined using small-area level information on household income, education attainment, and unemployment rate, and linked with individual data using Global Positioning System-coordinates. Associations between neighbourhood disadvantage and co-occurrence of three behaviour-related risk factors (smoking, heavy alcohol use, and physical inactivity), and the extent to which individual and neighbourhood level covariates explain neighbourhood differences in co-occurrence of risk factors were determined with multilevel cumulative logistic regression.

**Results:**

After adjusting for age, sex, marital status, and population density we found a dose-response relationship between neighbourhood disadvantage and co-occurrence of risk factors within each level of individual socioeconomic status. The cumulative odds ratios for the sum of health risks comparing the most to the least disadvantaged neighbourhoods ranged between 1.13 (95% confidence interval (CI): 1.03–1.24) and 1.75 (95% CI, 1.54–1.98). Individual socioeconomic characteristics explained 35%, and neighbourhood disadvantage and population density 17% of the neighbourhood differences in the co-occurrence of risk factors.

**Conclusions:**

Co-occurrence of poor health behaviours associated with neighbourhood disadvantage over and above individual's own socioeconomic status. Neighbourhood differences cannot be captured using individual socioeconomic factors alone, but neighbourhood level characteristics should also be considered.

## Introduction

Several studies have found associations between neighbourhood socioeconomic characteristics and behaviour-related risk factors [1], [2], [3], [4], [5], [6], [7], [8], [9], suggesting that neighbourhood characteristics may influence health behaviours of individuals, and that individuals' choices regarding residential areas may be associated with behaviour-related factors [10]. There is, for example, cross-sectional evidence to suggest that inadequate physical activity is associated with neighbourhood deprivation [4], [6], [7]. In addition, smoking prevalence, as well as excess alcohol intake, is often higher among those living in deprived neighbourhoods compared to those living in wealthier areas [5], [7], [11].

Despite the wide range of studies on individual health risks and health behaviours, very few studies on neighbourhoods have taken into account the effects on the accumulation of risk behaviours [9]. Risk behaviours tend to cluster within individuals [12], particularly among disadvantaged groups [13], [14], and some of this clustering may also be linked to shared neighbourhood characteristics. Moreover, in prior studies the neighbourhood differences have scarcely been quantified using measures familiar to researchers [15]. Thus, the extent to which neighbourhood level covariates can explain individual behaviours is not clear.

In this study, we hypothesized that the link between neighbourhood disadvantage and co-occurrence of poor health behaviours would be evident within each level of individual socioeconomic status, and these associations were studied by three individual level socioeconomic factors: occupational position, residence size, and residence ownership. We also quantified the extent to which individual and neighbourhood level covariates can explain the neighbourhood differences in co-occurrence of poor health behaviours.

## Methods

### Ethics statement

The ethics committee of Helsinki and Uusimaa Hospital District has approved the study. Written consents were not needed as the analyses were performed anonymously using research identification codes.

### Study population

Data are from the Finnish Public Sector Study, an ongoing prospective study among employees working in 10 towns and six hospital districts [16]. The register cohort compose of all employees employed by the target organizations for more than six months in any year between 1991 and 2005 (n = 151 618). Employers' records have been used to identify the eligible employees for a nested survey cohort; questionnaire surveys have been repeated every four years, starting from year 2000.

**Figure 1:**
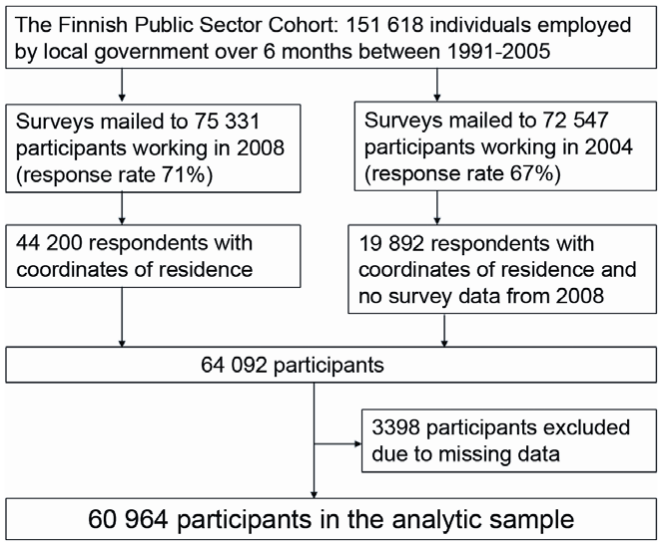
Flow chart describing selection of study participants for the analyses.

In this study, cross-sectional data on behaviour-related factors from the two most recent surveys were used. First, all employed cohort members responding to the 2008 survey and with coordinate data of their home address were included (n = 44 200), this population then, was completed with those from the 2004 survey (n = 19 892) if a participant had left the organization before 2008 or did not respond to the survey in 2008 (Figure 1). Missing data in the questionnaires were replaced with data from the surveys of years 2004 and 2000 when possible, but 3398 participants were excluded due to missing data. This analytic sample of 60 694 participants did not differ substantially from the eligible population in terms of mean age (47.2 years in the sample versus 47.1 years in the eligible population) or the proportion of women (80.0% vs. 76.9%), and those in the low occupational position (16.1% vs. 18.3%). The home addresses on the date of the survey and the Global Positioning System (GPS)-coordinates of these addresses were obtained from the Population Register Centre and successfully linked to the eligible population.

### Neighbourhoods

For the definition and characterization of neighbourhoods we used a grid database that contains Statistics Finland's coordinate based statistical data calculated by map grid [17]. It covers data describing the structure of the population including information on education, main type of activity, and household income within 250×250 m and 1×1 km squared areas. A 250×250 m square defined a neighbourhood in this study. The demographic grid data were based on the total population in Finland and were collected in 2008–2009. The questionnaire data were linked to the neighbourhoods using the Global Positioning System -coordinates.

We calculated summary scores for the socioeconomic environments of each neighbourhood using the grid database information on income (median household income in the area logarithmically transformed and then coded as additive inverse in order to obtain higher values for greater deprivation), education attainment (percentage of adults aged >18 years whose highest education level is elementary school), and unemployment rate (unemployed persons belonging to the labour force/total labour force). Income, education and unemployment are the most standard variables, either separately or jointly, used to characterize neighbourhood disadvantage and deprivation [18], [19]. For each of the three variables, we derived a standardized z-score (mean = 0, standard deviation = 1). Neighbourhood socioeconomic disadvantage scores were then calculated by taking the mean value across all z-scores [18], [19] when the z-score for at least one of the indicators was available. Lower summary scores indicate lower neighbourhood disadvantage. Missing data for the 250×250 m neighbourhoods (i.e. information on income and education was confidential if <10 cases within a square at the time of demographic data collection), were replaced using information from the eight surrounding neighbourhoods (750×750 m, for 3556 participants in 3323 neighbourhoods). The means of the indicator variables in the surrounding neighbourhoods were used for calculating the z-scores as well as the summary score for neighbourhood disadvantage. Another area level covariate derived from the grid database was population density (inhabitants/km^2^) that was used as a proxy for the degree of urbanization.

### Behaviour-related risk factors

We assessed three behaviour-related risk factors using standard questionnaire measurements in the postal surveys. In the surveys we inquired about smoking status (current vs. not), and the habitual frequency and amount of beer, wine, and spirits intake, which was transformed into grams of alcohol per week. One unit of pure alcohol (12 g) was equal to a 12 cl glass of wine, a 4 cl measure of spirits or a 33 cl bottle of beer. Heavy alcohol use was determined as >24 and >16 units per week for men and women, respectively. These limits correspond to the lower limits of heavy alcohol use by the Finnish Ministry of Social Affairs [20] as well as the medium risk levels of daily consumption of 41–60 g and 21–40 g per day for men and women, respectively, set by the World Health Organization [21]. Physical activity was measured by the Metabolic Equivalent Task (MET) index and was expressed as the summed score of Metabolic Equivalent Task hours per day.

The main outcome variable was a sum of the three dichotomized behaviour-related risk factors: 1) being a current smoker, 2) heavy alcohol use, and 3) leisure-time physical inactivity (<2.0 Metabolic Equivalent Task hours per day, corresponding to approximately 30 minutes of brisk walking [22]).

### Individual characteristics

Occupational position was used as one indicator of individual level socioeconomic status. As in our earlier studies [16] we derived participants' occupational titles (based on the International Standard Classification of Occupations [ISCO-88] [23]) from employers' administrative records. We then used the Classification of Occupations by Statistics Finland [24], an established classification system, to classify individuals into three occupational positions: the high = upper grade non-manual workers (professionals e.g. teachers, physicians), intermediate = lower grade non-manual workers (technicians and assistant professionals e.g. registered nurses), and the low = manual workers (service and care workers e.g. cleaners, maintenance and agricultural workers). This classification is based on the activities performed in the job and has previously been used in our studies; however, it may not be identical to those used in other studies. As we had no information on the income of the participants, the size of the residence and residence ownership were used as other indicators of individual socioeconomic status [25], [26]. Data on residence sizes (m^2^) and ownership (owner vs. not) were obtained from the Population Register Centre. The indicator for socioeconomic status by residence size was categorised as: high (>100 m^2^), intermediate (70–100 m^2^) and low (<70 m^2^), and by residence ownership as: high (owner) and low (not owner). Information about the study participants' age and sex was obtained from the employers' records, and marital status was assessed in the surveys.

Socioeconomic status is associated with the risk of mortality [27], [28], and our indicators of socioeconomic status also associated with mortality. During a 10-year follow-up in this cohort of public sector employees, the age and sex adjusted hazard ratio for mortality in the low vs. high occupational position was 1.83 (95% CI 1.67–2.01), among those living in the small vs. large residence 2.20 (95% CI 2.02–2.39), and among non residence owners vs. owners 1.62 (95% CI 1.51–1.73). These observations suggest the used indicators are valid proxies for individual socioeconomic status.

### Statistical analyses

The main outcome variable (i.e. the cumulative risk score for smoking, heavy alcohol use, and physical inactivity) used in the analyses had four classes (0, 1, 2, and 3 risks). To examine the association between neighbourhood disadvantage and the cumulative risk score, we tested whether the proportional odds assumption held (Score Test for the Proportional Odds Assumption), and used two-level cumulative logistic regression (GLIMMIX procedure of SAS 9.2) [29] that accounts for possible clustering of individuals within the neighbourhoods. First we examined possible interaction between sex and neighbourhood disadvantage by including term “disadvantage×sex” into a logistic regression model. For the main results we calculated cumulative odds ratios (COR) and their 95% confidence intervals (CI) by quintiles of neighbourhood disadvantage and by individual socioeconomic status (occupational position, residence size, and residence ownership, analyzed separately). The estimated cumulative odds ratio is an average of three specific logistic comparisons: ≥1 vs. <1 risk, ≥2 vs. <2 risks and 3 vs. <3 risks. In these analyses, the lowest quintile of neighbourhood disadvantage within the socioeconomic group was used as the reference and the models were adjusted for age, sex, marital status and population density. We ran also models adjusted for a regional unit “county”, but as this covariate had no effect on the results, and to keep the model simple, it was not included in the final analyses.

To examine the fixed effects of individual and neighbourhood level covariates on the risk sum, and the extent to which these variables explain the random neighbourhood effects, we ran four model specifications. We started with “model 1” including only a random intercept for neighbourhood in order to detect the existence of the possible contextual effect. Then we gradually added the fixed variables to the model: age, sex, and marital status (model 2), individual socioeconomic characteristics indicated by occupational position, residence size and residence ownership (model 3), and population density and neighbourhood disadvantage (model 4). The fixed effects are presented as CORs (95% CI), and the random effects as neighbourhood variance with standard error (SE). The variance is also translated into the median odds ratio (MOR), i.e.the neighbourhood-level variance in the odds ratio scale that enables better comparison between the magnitudes of the fixed and random effects [15], [30]. MOR quantifies the variation between neighbourhoods (the second-level variation) by comparing two persons from two randomly chosen neighbourhoods, i.e., the MOR is the median odds ratio between the person of higher propensity and the person of lower propensity. Values of MOR are always ≥1, if it is 1 there is no neighbourhood level variation.

As sensitivity analyses, we ran the adjusted models 1) excluding areas that were represented by only one participant, and 2) using 1×1 km definition for a neighbourhood. We also tested whether some of the variables of the neighbourhood index drive the results by analyzing separately each index component. To estimate whether time of residence in the neighbourhood affects the results, we tested the interaction between living more or less than five years in the neighbourhood and neighbourhood disadvantage.

## Results

The mean age of participants was 47.2 years (range 18–72). A majority of the study participants held intermediate occupational position (51.8%), and owned their residence (71.1%), one third (34.9%) lived in medium sized residence (70 to 100 m^2^). The 60 964 participants lived in 18 704 neighbourhoods and the number of study participants within neighbourhoods ranged from 1 to 134. The mean number of participants per neighbourhood was 11 (SE = 12). Of the participants 15.1% were the only person representing their neighbourhood. More detailed descriptive data on the study participants and neighbourhood characteristics by quintiles of neighbourhood disadvantage are presented in Table 1.

**Table 1:** Descriptive data of the study participants and neighbourhood characteristics by quintiles of neighbourhood disadvantage.

	Neighbourhood disadvantage				
	Q1 (lowest)	Q2	Q3	Q4	Q5 (highest)
Individual level variable	n of participants (%)				
Socioeconomic status by:					
Occupational position	12116	12191	12197	12161	12029
High (upper grade non-manual)	4988 (41.2)	4508 (37.0)	4177 (34.2)	3367 (27.7)	2419 (20.1)
Intermediate (lower grade non-manual)	5814 (48.0)	6206 (50.9)	6219 (51.0)	6632 (54.5)	6592 (54.8)
Low (manual)	1314 (10.8)	1477 (12.1)	1801 (14.8)	2162 (17.8)	3018 (25.1)
Residence size					
High (>100 m^2^)	7789 (64.3)	5805 (47.6)	4391 (36.0)	2976 (24.5)	2433 (20.2)
Intermediate (70–100 m^2^)	3153 (26.0)	4058 (33.3)	4405 (36.1)	4646 (38.2)	4921 (40.9)
Low (<70 m^2^)	1174 (9.7)	2328 (19.1)	3401 (27.9)	4539 (37.3)	4675 (38.9)
Residences ownership					
High (own)	7202 (83.2)	6350 (75.7)	6061 (71.7)	5532 (65.7)	4581 (58.3)
Low (not own)	1453 (16.8)	2040 (24.3)	2387 (28.3)	2893 (34.3)	3275 (41.7)
Current smoker					
Yes	1290 (10.7)	1530 (12.6)	1781 (14.6)	2106 (17.3)	2590 (21.5)
Heavy alcohol use (>24 or >16 units per week)					
Yes	990 (8.2)	1046 (8.6)	1029 (8.4)	1066 (8.8)	971 (8.1)
Physical inactivity (<2 Metabolic Equivalent Task hours/day)					
Yes	2845 (23.5)	2942 (24.1)	2977 (24.4)	3122 (25.7)	3560 (29.6)
Married or cohabiting					
Yes	10 731 (88.6)	9916 (81.3)	9119 (74.8)	8297 (68.2)	7695 (64.0)
Risk sum (smoking, heavy alcohol use, physical inactivity)					
0	7782 64.2)	7621 (62.5)	7486 (61.384)	7079 (58.22)	6392 (53.2)
1	3612 (29.8)	3707 (30.4)	3743 (30.7)	3983 (32.8)	4276 (35.6)
2	648 (5.4)	774 (6.35)	860 (7.05)	984 (8.09)	1223 (10.2)
3	72 (0.59)	86 (0.71)	108 (0.89)	114 (0.94)	132 (1.10)
Neighbourhood variable	Mean (standard error)				
Median annual household income, €	68 051 (17 056)	52 927 (13 191)	42 945 (12 353)	35 033 (10 476)	25 951 (10 213)
Proportion of low education, %	13.2 (5.1)	19.8 (5.1)	24.2 (5.4)	29.4 (6.5)	38.1 (8.8)
Unemployment rate, %	1.9 (2.1)	4.4 (2.6)	6.4 (2.8)	9.0 (3.6)	15.5 (7.3)
Population density	1820 (2375)	3006 (3419)	4345 (4395)	5229 (4646)	4887 (4259)

The interaction between sex and neighbourhood disadvantage was non-significant (p-value 0.45) suggesting no differences by sex, thus, all analyses were conducted for women and men combined. The conservative test for the proportional odds assumption for the cumulative regression was not fulfilled. However, the large size of the data may have resulted in statistical significance of the test score; thus, we further investigated the assumption by comparing results of multinomial and cumulative logistic regression analyses. These results were comparable (File S1) and therefore we present results for the simpler ordinal model.

**Figure 2:**
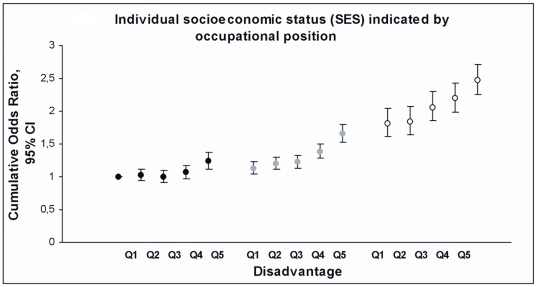
Co-occurrence of risk factors by quintiles of neighbourhood disadvantage and by individual socioeconomic status. Cumulative Odds Ratios (COR, i.e. the average of three specific logistic comparisons: ≥1 vs. <1 risk, ≥2 vs. <2 risks and 3 vs. <3 risks) and 95% Confidence Intervals (CI) from models where the lowest quintile of disadvantage in the high socioeconomic status group is the reference for all groups.

**Figure 3:**
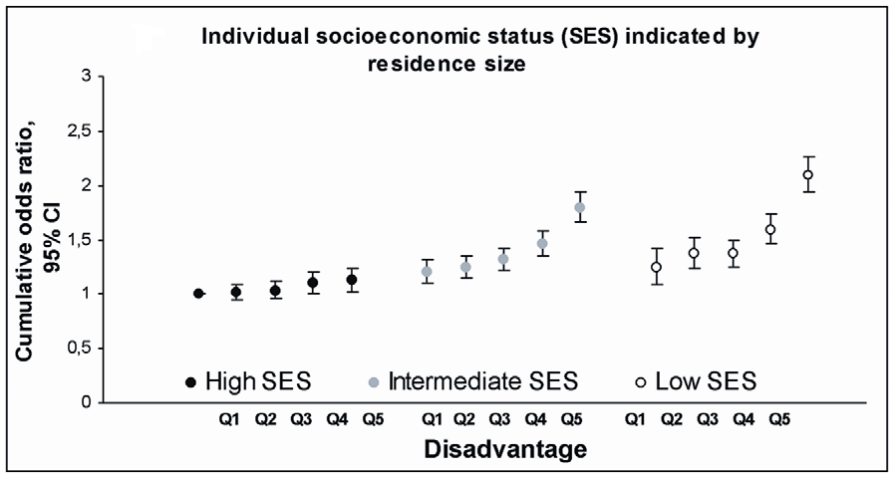
Co-occurrence of risk factors by quintiles of neighbourhood disadvantage and by individual socioeconomic status. Cumulative Odds Ratios (COR, i.e. the average of three specific logistic comparisons: ≥1 vs. <1 risk, ≥2 vs. <2 risks and 3 vs. <3 risks) and 95% Confidence Intervals (CI) from models where the lowest quintile of disadvantage in the high socioeconomic status group is the reference for all groups.

**Figure 4:**
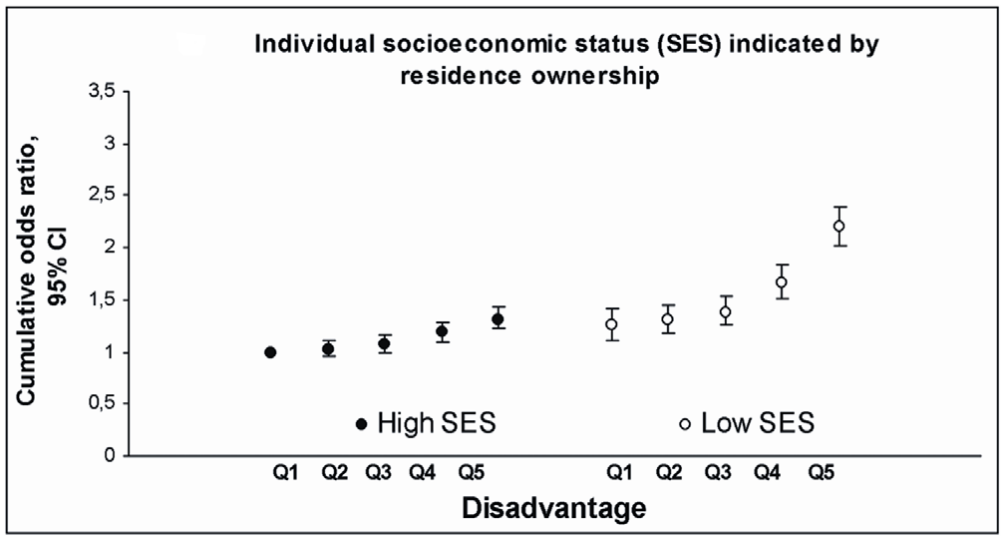
Co-occurrence of risk factors by quintiles of neighbourhood disadvantage and by individual socioeconomic status. Cumulative Odds Ratios (COR, i.e. the average of three specific logistic comparisons: ≥1 vs. <1 risk, ≥2 vs. <2 risks and 3 vs. <3 risks) and 95% Confidence Intervals (CI) from models where the lowest quintile of disadvantage in the high socioeconomic status group is the reference for all groups.

We found that within each socioeconomic group, there was a growing trend in the likelihood of multiple risk behaviours with increasing neighbourhood disadvantage. The graded associations between neighbourhood adversity and risk behaviours by occupational positions, residence size, and residence ownership are shown in Figures 2, 3, and 4 (where individuals of the most favourable group residing in the least disadvantaged areas were used as the reference). Interestingly, those occupying the intermediate socioeconomic status and living in the least or second least disadvantaged area had lower cumulative odds ratios (e.g. for intermediate occupational position: COR 1.09, 95% CI 1.01–1.18) than those with high socioeconomic status but living in the most disadvantaged neighbourhood (high occupational position: COR 1.35, 95% CI 1.21–1.49) (File S1). Within socioeconomic groups, measured by occupational position, residence size, and residence ownership, the cumulative odds ratios for risk behaviours, i.e. the average likelihood of having ≥1 vs. <1, ≥2 vs. <2, or 3 vs. <3 risk factors, ranged between 1.13 (95% CI, 1.03–1.24) and 1.75 (95% CI, 1.54–1.98) (File S1) if living in the most compared to the least disadvantaged neighbourhood.

The relative effects of the fixed covariates on the risk sum showed that all covariates associated with the co-occurrence of risk factors (Table 2). Between neighbourhood variance in the co-occurrence of risk behaviours was also significant in all model specifications with median odds ratio quantifying the differences ranging from 1.29 to 1.20 (Table 2). Individual demographic characteristics explained little (3.5%) of the neighbourhood variance, whereas individual level socioeconomic variables explained 35% of the variance. Controlling for individual level covariates, neighbourhood disadvantage and area level population density explained another 17% of the between neighbourhood variance in the co-occurrence of risks, however, median odds ratio of 1.20 shows that some variation remained unexplained. A post hoc analysis including individual variables only for sex and age, and the two area level variables, showed that neighbourhood disadvantage and population density explained same amount (18%) of the between neighbourhood variance as the more adjusted model.

**Table 2:** Fixed effects of the covariates and random effects of neighbourhood from the two-level cumulative regression analyses.

	Model^a^			Model^b^			Model^c^			Model^d^		
Fixed Part	COR	95% CI		COR	95% CI		COR	95% CI		COR	95% CI	
Age (per 10 years)				1.13	1.11	1.15	1.12	1.10	1.1491	1.12	1.10	1.15
Sex (male vs. female)				1.87	1.80	1.95	1.75	1.66	1.84	1.75	1.67	1.84
Marital status (single vs. cohabiting)				1.30	1.25	1.35	1.08	1.03	1.14	1.07	1.02	1.12
Socioeconomic status by:												
Occupational position												
High (ref)							1			1		
Intermediate							1.23	1.18	1.29	1.21	1.16	1.27
Low							1.81	1.70	1.92	1.75	1.64	1.86
Size of residence												
High (ref)							1			1		
Intermediate							1.25	1.19	1.31	1.23	1.17	1.29
Low							1.31	1.23	1.38	1.28	1.20	1.36
Residence ownership												
High (ref)							1			1		
Low							1.33	1.27	1.39	1.31	1.25	1.37
Population density										0.98	0.96	1.00
Neighbourhood disadvantage												
Q1										1		
Q2										0.99	0.93	1.06
Q3										1.00	0.94	1.07
Q4										1.09	1.02	1.17
Q5										1.26	1.17	1.34
Random Part												
Neighbourhood												
Variance (SE)	0.0729 (0.009)*			0.0704 (0.009)*			0.0457 (0.011)*			0.0382 (0.011)*		
Proportional chance in variance (%)	-			−3.5			−35.0			−16.6		
Median odds ratio (MOR)^e^	1.29			1.29			1.23			1.20		

a Crude model,

b Model adjusted for age, sex, and marital status,

c Model b adjusted for occupational position, size of residence, and residence ownership,

d Model c adjusted for population density and neighbourhood disadvantage,

e increased risk that (in median) one would have if moving to a neighbourhood with a higher risk,

* P-value<0.001.

In the sensitivity analysis excluding areas that were represented by only one participant, the results for the co-occurrence of risk factors remained mainly unchanged (File S1), and the proportion of the outcome variance explained by neighbourhood characteristics remained the same (17%). When 1×1 km neighbourhood definition was used the associations were weaker and less gradual compared to those when using the definition of 250×250 m (File S1). We also found that population density and neighbourhood disadvantage aggregated to 1×1 km squares explained 2.8% of the variance in co-occurrence of risk factors. Interaction between living in the neighbourhood less or more than five years and neighbourhood disadvantage was not significant (p-value 0.77), which suggests that time of residence does not have influence on the observed associations. No single aspect of the neighbourhood index variables was responsible for driving the results (File S1).

## Discussion

In this study of over 60 000 public sector employees, we found that living in a disadvantaged neighbourhood was associated with co-occurrence of risk behaviours - smoking, heavy alcohol use, and physical inactivity - regardless of individual socioeconomic circumstances. We also calculated that neighbourhood disadvantage and area level population density explained almost a fifth of the neighbourhood differences in the clustering of these risk behaviours after controlling for individual factors. This suggests that neighbourhood differences cannot be explained using individual level socioeconomic factors alone, but neighbourhood level covariates should also be considered.

Prior evidence about the neighbourhood effects on clustering of risk factors is very limited [9] as most studies have reported results for single health risks. We found evidence that neighbourhood disadvantage is, in addition to and regardless of individual socioeconomic characteristics [13], [14], related to clustering of behaviour-related risk factors. We even found evidence for a hypothesis that people in the lower socioeconomic groups may benefit from living in more advantaged areas. Although individual socioeconomic factors explained also over a third of the between neighbourhood variance in the co-occurrence of risks, these factors did not capture the whole picture. We found that individuals with risky behaviours are not clustered into neighbourhoods only with respect to their demographic or socioeconomic characteristics, but that the co-occurrence of risk behaviours depends also on the clustering of neighbourhood-level disadvantage measured using small spatial units. Larger spatial unit resulted in attenuated effect of neighbourhoods, which suggests large spatial units may not capture local variation in the area level variables as well as the small one. Even though part of the between neighbourhood variance remained unexplained in this study, these findings suggest that controlling only for individual level socioeconomic factors is not an adequate way to account for neighbourhood level socioeconomic differences in epidemiological studies. However, further research is also needed to determine what additional factors are related to these neighbourhood differences.

There are several possible pathways through which neighbourhood disadvantage may influence behaviour-related risks. One is that disadvantaged neighbourhoods may not facilitate wide social networks, social cohesion, social support and sense of belonging [31], [32], and achieving these features may be difficult if social resources (e.g. opportunities for cultural and social activities and access to information) are lacking in the same neighbourhoods [8]. A recent study suggested that in disadvantaged neighbourhoods individuals are more exposed to poor health behaviours than affluent peers, which may influence the initiation and maintenance of healthy behaviours [33]. There may also be social stressors such as high crime or traffic rates in the deprived neighbourhoods that influence multiple health behaviours, for instance, by increasing the prevalence of smoking or limiting physical activities [2], [34]. Specifically, if the stressors are simultaneously present in the neighbourhoods, their multiplicative effects on health behaviours are likely.

The structure of the neighbourhood may also play a role in the association between neighbourhood disadvantage and behaviour-related risks. In the U.S., for example, the lack of facilities for sports and leisure services, sidewalks or bike paths [35] have been suggested as possible reasons for the increased likelihood of sedentary lifestyle, while higher level of alcohol consumption has been related to greater alcohol outlet density in the neighbourhood [36]. Disadvantaged neighbourhoods often concurrently suffer from multiple structural shortcomings which may increase the likelihood of co-occurrence of poor health behaviours. In Finland, grocery stores in the disadvantaged neighbourhoods are often small and therefore likely to offer few choices of vegetables and other healthy foods; however, even in the smallest stores tobacco products and beer are sold, which in turn may increase the prevalence of smoking and heavy alcohol use. This suggests that the quality of the services is also of importance. The observed neighbourhood effects, and the various possible reasons for them, suggest there are many issues policy makers could take into account when aiming at reducing socioeconomic differences between neighbourhoods.

This study has marked strengths but also some limitations. The use of self-reported data may have resulted in bias, as respondents may have under-reported their risk behaviours. Self-reporting tends to under-estimate smoking [37] and alcohol use [38] in the population. These may have under- or overestimated the effects if underestimations were socially patterned. However, the same-source bias was avoided by using self-reports and grid database information. Despite the lack of consensus on whether perceived or objective measures are more valid for defining neighbourhoods, objective characteristics of small neighbourhoods - such as 250×250 m squares used in this study - may correlate well with how residents define their neighbourhood. In addition, we used an index variable for defining neighbourhood disadvantage including area-level median household income, unemployment rate and education attainment, variables widely used when assessing socioeconomic status of a neighbourhood. Our approach may have masked some variation as areas with the same disadvantage score may have had different values contributing to the score [39]. However, the facts that no single variable in the index was driving the results, and that all three variables exhibited variation between neighbourhood disadvantage quintiles provide support to the use of the index variable. Because the study design was cross-sectional we are limited in our ability to draw causal inferences. It is possible, for example, that areas where alcohol addicts and heavy smokers are clustered become less attractive, which encourages people to move somewhere else. However, choosing the place of residence may only be possible for wealthy people who might choose to live elsewhere, which can lead to lower socioeconomic status for the unattractive neighbourhood. Finally, as our study sample consisted of public sector employees in a Scandinavian welfare country, the generalizability of the results to countries with large variation in area disadvantage, or to general non-employed populations, remains unclear.

In summary, we found evidence to suggest that living in a more disadvantaged neighbourhood increase the likelihood of co-occurrence of unhealthy behaviours at all levels of individual socioeconomic status. Our results also suggest that neighbourhood differences cannot be captured by controlling for individual socioeconomic characteristics, but neighbourhood level characteristics need to be considered as well.

## Supporting Information

File S1 (DOC)
