# Supplementary material for: Quantifying Neighbourhood Socioeconomic Effects in Clustering of Behaviour-Related Risk Factors: A Multilevel Analysis
Source: PLoS One. 2012 Mar 12;7(3):e32937. doi: 10.1371/journal.pone.0032937 (PMC3299718; doi:10.1371/journal.pone.0032937)
Supplement: File S1 — (DOC) [file pone.0032937.s001.doc]

**Supporting information S1**

**Halonen et al.**

"Quantifying neighbourhood socioeconomic effects in clustering of behaviour-related risk factors: a multilevel analysis"

**Table S1.** Comparison of results from multinomial and cumulative regression analyses using crude model for the association between neighbourhood disadvantage and sum of health-related behaviors for the total study population.

| **Neighbourhood Disadvantage** | **Multinomial logistic regression** | | | | | | | | | **Cumulative regression** | | |
| --- | --- | --- | --- | --- | --- | --- | --- | --- | --- | --- | --- | --- |
|  | **1 vs. 0 risk** | | | **2 vs. 0 risks** | | | **3 vs. 0 risks** | | |  | | |
|  | OR | 95% CI | | OR | 95% CI | | OR | 95% CI | | COR | 95% CI | |
| Q1 | 1 |  |  | 1 |  |  | 1 |  |  | 1 |  |  |
| Q2 | 1.05 | 0.99 | 1.11 | 1.22 | 1.09 | 1.36 | 1.22 | 0.89 | 1.67 | 1.08 | 1.03 | 1.14 |
| Q3 | 1.08 | 1.02 | 1.14 | 1.38 | 1.24 | 1.54 | 1.56 | 1.16 | 2.10 | 1.15 | 1.09 | 1.22 |
| Q4 | 1.21 | 1.14 | 1.28 | 1.67 | 1.50 | 1.85 | 1.74 | 1.29 | 2.34 | 1.31 | 1.24 | 1.38 |
| Q5 | 1.44 | 1.36 | 1.52 | 2.29 | 2.06 | 2.53 | 2.23 | 1.67 | 2.98 | 1.62 | 1.53 | 1.70 |

**Table S2**. Cumulative odds ratios (COR) and 95% Confidence Intervals (CI) for the associations between neighborhood disadvantage and the sum of behavior-related risk factors by occupational position.

| **Risk sum ^a^** | | | | | | | | | |
| --- | --- | --- | --- | --- | --- | --- | --- | --- | --- |
| Neighborhood disadvantage | **Original model** | | | **The only representatives of area excluded** | | | **1 x 1 km neighbourhood definition** | | |
|  | COR | 95% CI | | COR | 95% CI | | COR | 95% CI | |
| High occupational position | | | |  |  |  |  |  |  |
| Q1 (lowest) | 1 |  |  | 1 |  |  | 1 |  |  |
| Q2 | 1.01 | 0.92 | 1.10 | 1.03 | 0.93 | 1.13 | 0.96 | 0.88 | 1.05 |
| Q3 | 1.04 | 0.95 | 1.14 | 1.06 | 0.96 | 1.17 | 1.13 | 1.03 | 1.23 |
| Q4 | 1.10 | 0.99 | 1.21 | 1.13 | 1.02 | 1.26 | 1.05 | 0.96 | 1.15 |
| Q5 (highest) | 1.35 | 1.21 | 1.49 | 1.37 | 1.22 | 1.54 | 1.05 | 0.95 | 1.16 |
| Intermediate occupational position | | | |  |  |  |  |  |  |
| Q1 (lowest) | 1 |  |  | 1 |  |  | 1 |  |  |
| Q2 | 1.09 | 1.01 | 1.18 | 1.12 | 1.02 | 1.22 | 1.01 | 0.93 | 1.08 |
| Q3 | 1.10 | 1.02 | 1.19 | 1.14 | 1.05 | 1.25 | 1.07 | 0.99 | 1.15 |
| Q4 | 1.25 | 1.16 | 1.35 | 1.30 | 1.19 | 1.42 | 1.09 | 1.01 | 1.17 |
| Q5 (highest) | 1.50 | 1.39 | 1.62 | 1.57 | 1.44 | 1.71 | 1.23 | 1.15 | 1.33 |
| Low occupational position | | | |  |  |  |  |  |  |
| Q1 (lowest) | 1 |  |  | 1 |  |  | 1 |  |  |
| Q2 | 1.08 | 0.93 | 1.25 | 1.04 | 0.88 | 1.22 | 1.17 | 1.02 | 1.34 |
| Q3 | 1.22 | 1.06 | 1.40 | 1.18 | 1.01 | 1.38 | 1.09 | 0.95 | 1.25 |
| Q4 | 1.28 | 1.12 | 1.47 | 1.21 | 1.04 | 1.41 | 1.16 | 1.01 | 1.32 |
| Q5 (highest) | 1.54 | 1.36 | 1.75 | 1.52 | 1.32 | 1.76 | 1.23 | 1.08 | 1.40 |

^a^ Model adjusted for age, sex, marital status and population density

**Table S3**. Cumulative odds ratios (COR) and 95% Confidence Intervals (CI) for the sum associations between neighborhood disadvantage and the sum of behavior-related risk factors by residence size.

| **Risk sum ^a^** | | | | | | | | | | | | | | |  |
| --- | --- | --- | --- | --- | --- | --- | --- | --- | --- | --- | --- | --- | --- | --- | --- |
| **Neighborhood disadvantage** | **Original** | | | **The only representatives of area excluded** | | | | **1 x 1 km neighbourhood definition** | | | | | | |  |
|  | COR | 95% CI | | | COR | 95% CI | | | COR | | 95% CI | | | | |
| Large residence size | |  | | |  |  | | |  | |  | | | | |
| Q1 (lowest) | 1 |  |  | | 1 |  |  | | 1 | |  | |  | | |
| Q2 | 1.02 | 0.95 | 1.09 | | 1.04 | 0.96 | 1.13 | | | 0.98 | | 0.91 | | 1.06 | |
| Q3 | 1.04 | 0.96 | 1.12 | | 1.06 | 0.97 | 1.16 | | | 1.09 | | 1.00 | | 1.18 | |
| Q4 | 1.11 | 1.01 | 1.21 | | 1.11 | 1.00 | 1.23 | | | 0.99 | | 0.91 | | 1.09 | |
| Q5 (highest) | 1.13 | 1.03 | 1.24 | | 1.08 | 0.96 | 1.22 | | | 1.03 | | 0.94 | | 1.13 | |
| Intermediate residence size | |  |  | |  |  |  | | |  | |  | |  | |
| Q1 (lowest) | 1 |  |  | | 1 |  |  | | | 1 | |  | |  | |
| Q2 | 1.03 | 0.94 | 1.14 | | 1.02 | 0.92 | 1.13 | | | 1.08 | | 0.99 | | 1.19 | |
| Q3 | 1.09 | 0.99 | 1.20 | | 1.10 | 0.99 | 1.22 | | | 1.10 | | 1.00 | | 1.20 | |
| Q4 | 1.21 | 1.10 | 1.33 | | 1.22 | 1.10 | 1.35 | | | 1.18 | | 1.08 | | 1.30 | |
| Q5 (highest) | 1.49 | 1.36 | 1.63 | | 1.50 | 1.36 | 1.66 | | | 1.32 | | 1.20 | | 1.45 | |
| Small residence size | |  |  | |  |  |  | | |  | |  | |  | |
| Q1 (lowest) | 1 |  |  | | 1 |  |  | | | 1 | |  | |  | |
| Q2 | 1.10 | 0.95 | 1.28 | | 1.15 | 0.98 | 1.35 | | | 1.02 | | 0.89 | | 1.16 | |
| Q3 | 1.10 | 0.96 | 1.27 | | 1.14 | 0.97 | 1.33 | | | 1.14 | | 1.00 | | 1.29 | |
| Q4 | 1.28 | 1.12 | 1.47 | | 1.34 | 1.16 | 1.56 | | | 1.10 | | 0.98 | | 1.24 | |
| Q5 (highest) | 1.68 | 1.48 | 1.92 | | 1.77 | 1.52 | 2.05 | | | 1.29 | | 1.14 | | 1.46 | |

^a^ Model adjusted for age, sex, marital status, and population density

**Table S4**. Cumulative odds ratios (COR) and 95% Confidence Intervals (CI) for the sum associations between neighborhood disadvantage and the sum of behavior-related risk factors by residence ownership.

| **Risk sum ^a^** | | | | | | | | | | | | | | |  |
| --- | --- | --- | --- | --- | --- | --- | --- | --- | --- | --- | --- | --- | --- | --- | --- |
| **Neighborhood disadvantage** | **Original** | | | **The only representatives of area excluded** | | | | **1 x 1 km neighbourhood definition** | | | | | | |  |
|  | COR | 95% CI | | | COR | 95% CI | | | COR | | 95% CI | | | | |
| Own residence | |  | | |  |  | | |  | |  | | | | |
| Q1 (lowest) | 1 |  |  | | 1 |  |  | | 1 | |  | |  | | |
| Q2 | 1.03 | 0.96 | 1.11 | | 1.04 | 0.96 | 1.13 | | | 1.05 | | 0.98 | | 1.13 | |
| Q3 | 1.07 | 1.00 | 1.16 | | 1.09 | 1.00 | 1.18 | | | 1.13 | | 1.04 | | 1.21 | |
| Q4 | 1.19 | 1.10 | 1.28 | | 1.21 | 1.11 | 1.31 | | | 1.15 | | 1.06 | | 1.24 | |
| Q5 (highest) | 1.32 | 1.22 | 1.43 | | 1.34 | 1.22 | 1.47 | | | 1.23 | | 1.14 | | 1.33 | |
| Residence not own | |  |  | |  |  |  | | |  | |  | |  | |
| Q1 (lowest) | 1 |  |  | | 1 |  |  | | | 1 | |  | |  | |
| Q2 | 1.04 | 0.91 | 1.20 | | 1.07 | 0.92 | 1.24 | | | 1.02 | | 0.90 | | 1.15 | |
| Q3 | 1.10 | 0.97 | 1.26 | | 1.14 | 0.98 | 1.32 | | | 1.18 | | 1.04 | | 1.33 | |
| Q4 | 1.33 | 1.17 | 1.51 | | 1.38 | 1.20 | 1.59 | | | 1.12 | | 1.00 | | 1.27 | |
| Q5 (highest) | 1.75 | 1.54 | 1.98 | | 1.83 | 1.59 | 2.10 | | | 1.39 | | 1.23 | | 1.56 | |

^a^ Model adjusted for age, sex, marital status, and population density

**Table S5**. Cumulative odds ratios (COR) and 95% Confidence Intervals (CI) for the associations of neighborhood education level, household median income and unemployment rate with the sum of behavior-related risk factors by occupational status.

| **Risk sum ^a^** | **Predictor** | | | | | | | | | | |
| --- | --- | --- | --- | --- | --- | --- | --- | --- | --- | --- | --- |
| **Quintile of the predictor** | **Low level of education** | | | | **Household income (inverse)** | | | **Unemployment rate** | | | |
|  | COR | | 95% CI | | COR | 95% CI | | COR | | 95% CI | |
| High occupational position | | | |  |  |  |  | |  |  |  |
| Q1 (lowest) | | 1 |  |  | 1 |  |  | | 1 |  |  |
| Q2 | | 1.04 | 0.95 | 1.14 | 1.05 | 0.96 | 1.14 | | 0.96 | 0.88 | 1.04 |
| Q3 | | 1.12 | 1.03 | 1.23 | 0.99 | 0.91 | 1.09 | | 0.98 | 0.90 | 1.08 |
| Q4 | | 1.11 | 1.01 | 1.22 | 1.13 | 1.03 | 1.24 | | 0.99 | 0.90 | 1.09 |
| Q5 (highest) | | 1.29 | 1.17 | 1.43 | 1.17 | 1.06 | 1.29 | | 1.09 | 0.98 | 1.21 |
| Intermediate occupational position | | | | |  |  |  | |  |  |  |
| Q1 (lowest) | | 1 |  |  | 1 |  |  | | 1 |  |  |
| Q2 | | 1.11 | 1.02 | 1.20 | 1.06 | 0.99 | 1.15 | | 1.02 | 0.95 | 1.10 |
| Q3 | | 1.15 | 1.06 | 1.24 | 1.17 | 1.08 | 1.26 | | 1.03 | 0.96 | 1.11 |
| Q4 | | 1.24 | 1.15 | 1.34 | 1.36 | 1.26 | 1.47 | | 1.10 | 1.02 | 1.18 |
| Q5 (highest) | | 1.51 | 1.40 | 1.63 | 1.41 | 1.31 | 1.53 | | 1.32 | 1.23 | 1.42 |
| Low occupational position | | | |  |  |  |  | |  |  |  |
| Q1 (lowest) | | 1 |  |  | 1 |  |  | | 1 |  |  |
| Q2 | | 0.99 | 0.85 | 1.16 | 1.10 | 0.96 | 1.27 | | 1.05 | 0.92 | 1.21 |
| Q3 | | 1.06 | 0.92 | 1.23 | 1.20 | 1.04 | 1.38 | | 1.11 | 0.97 | 1.27 |
| Q4 | | 1.12 | 0.97 | 1.29 | 1.35 | 1.18 | 1.55 | | 1.11 | 0.98 | 1.26 |
| Q5 (highest) | | 1.26 | 1.10 | 1.43 | 1.41 | 1.23 | 1.62 | | 1.31 | 1.16 | 1.48 |

^a^ Model adjusted for age, sex, marital status, and population density

**Table S6.** Cumulative odds ratios (COR) and 95% Confidence Intervals (CI) for the associations of neighborhood education level, household median income and unemployment rate with sum of behavior-related risk factors by residence size.

| **Risk sum ^a^** | **Predictor** | | | | | | | | | | | | | | | | | | | | | | |
| --- | --- | --- | --- | --- | --- | --- | --- | --- | --- | --- | --- | --- | --- | --- | --- | --- | --- | --- | --- | --- | --- | --- | --- |
| **Quintile of the predictor** | **Low level of education** | | | | **Household income (inverse)** | | | | | | | **Unemployment rate** | | | | | | | | | | | |
|  | COR | 95% CI | | | | COR | | | 95% CI | | | | | | COR | | | 95% CI | | | | | |
| Large residence size | |  | | | | |  | |  | | | |  | | | | | |  | | | |  |
| Q1 (lowest) | 1 |  |  | | | 1 | | |  | |  | | | 1 | | |  | | |  | | |  |
| Q2 | 1.02 | 0.94 | 1.11 | | | 1.06 | | | 0.99 | | 1.14 | | | 0.97 | | | 0.90 | | | 1.05 | | |  |
| Q3 | 1.16 | 1.07 | 1.27 | | | 1.06 | | | 0.98 | | 1.14 | | | 0.97 | | | 0.90 | | | 1.06 | | |  |
| Q4 | 1.13 | 1.03 | 1.23 | | | 1.07 | | | 0.97 | | 1.19 | | | 1.01 | | | 0.93 | | | 1.09 | | |  |
| Q5 (highest) | 1.19 | 1.08 | 1.32 | | | 1.06 | | | 0.95 | | 1.19 | | | 1.08 | | | 0.99 | | | 1.18 | | |  |
| Intermediate residence size | |  | |  | | | |  | |  |  | | | | |  | | |  | | |  |  |
| Q1 (lowest) | 1 |  |  | | | 1 | | |  | |  | | |  | | |  | | |  | | |  |
| Q2 | 1.08 | 0.98 | 1.19 | | | 1.05 | | | 0.95 | | 1.16 | | | 1.03 | | | 0.94 | | | 1.13 | | |  |
| Q3 | 1.09 | 0.99 | 1.20 | | | 1.09 | | | 0.99 | | 1.20 | | | 1.07 | | | 0.98 | | | 1.18 | | |  |
| Q4 | 1.21 | 1.10 | 1.33 | | | 1.28 | | | 1.17 | | 1.41 | | | 1.13 | | | 1.03 | | | 1.24 | | |  |
| Q5 (highest) | 1.43 | 1.31 | 1.57 | | | 1.35 | | | 1.22 | | 1.49 | | | 1.38 | | | 1.25 | | | 1.51 | | |  |
| Low residence size | |  |  | | |  | | |  | |  | | | | |  | |  | | |  | |  |
| Q1 (lowest) | 1 |  |  | | | 1 | | |  | |  | | |  | | |  | | |  | | |  |
| Q2 | 1.15 | 1.01 | 1.30 | | | 1.07 | | | 0.90 | | 1.28 | | | 0.90 | | | 0.79 | | | 1.03 | | |  |
| Q3 | 1.20 | 1.06 | 1.35 | | | 1.10 | | | 0.93 | | 1.29 | | | 0.99 | | | 0.88 | | | 1.13 | | |  |
| Q4 | 1.33 | 1.18 | 1.50 | | | 1.24 | | | 1.06 | | 1.45 | | | 1.05 | | | 0.93 | | | 1.19 | | |  |
| Q5 (highest) | 1.74 | 1.55 | 1.95 | | | 1.36 | | | 1.17 | | 1.59 | | | 1.34 | | | 1.18 | | | 1.51 | | |  |

^a^ Model adjusted for age, sex, marital status, and population density

**Table S7.** Cumulative odds ratios (COR) and 95% Confidence Intervals (CI) for the associations of neighborhood education level, household median income and unemployment rate with sum of behavior-related risk factors by residence ownership.

| **Risk sum ^a^** | **Predictor** | | | | | | | | | | | | |
| --- | --- | --- | --- | --- | --- | --- | --- | --- | --- | --- | --- | --- | --- |
| **Quintile of the predictor** | **Low level of education** | | | **Household income (inverse)** | | | | **Unemployment rate** | | | | |  |
|  | COR | 95% CI | | | COR | 95% CI | | | COR | | 95% CI | | |
| Own residence | |  | | |  |  | | |  | |  | | |
| Q1 (lowest) | 1 |  |  | | 1 |  |  | | | 1 |  |  | |
| Q2 | 1.03 | 0.95 | 1.11 | | 1.14 | 1.06 | 1.22 | | | 0.97 | 0.90 | 1.04 | |
| Q3 | 1.13 | 1.04 | 1.22 | | 1.19 | 1.10 | 1.28 | | | 1.00 | 0.93 | 1.07 | |
| Q4 | 1.14 | 1.06 | 1.23 | | 1.31 | 1.21 | 1.42 | | | 1.05 | 0.98 | 1.14 | |
| Q5 (highest) | 1.37 | 1.26 | 1.48 | | 1.36 | 1.25 | 1.47 | | | 1.15 | 1.06 | 1.24 | |
| Not own residence | |  |  | |  |  |  | | |  |  |  | |
| Q1 (lowest) | 1 |  |  | | 1 |  |  | | |  |  |  | |
| Q2 | 1.14 | 0.99 | 1.30 | | 1.01 | 0.88 | 1.17 | | | 1.04 | 0.91 | 1.18 | |
| Q3 | 1.23 | 1.07 | 1.40 | | 1.14 | 1.00 | 1.30 | | | 1.10 | 0.97 | 1.26 | |
| Q4 | 1.46 | 1.29 | 1.66 | | 1.40 | 1.23 | 1.60 | | | 1.13 | 0.99 | 1.28 | |
| Q5 (highest) | 1.80 | 1.59 | 2.03 | | 1.55 | 1.36 | 1.76 | | | 1.60 | 1.42 | 1.82 | |

^a^ Model adjusted for age, sex, marital status and population density
